# Performance of GFAP and UCH-L1 for Early Acute Stroke Diagnosis in the Emergency Department

**DOI:** 10.3390/jcm14134746

**Published:** 2025-07-04

**Authors:** Daian-Ionel Popa, Florina Buleu, Aida Iancu, Anca Tudor, Carmen Gabriela Williams, Dumitru Sutoi, Adina Maria Marza, Cosmin Iosif Trebuian, Alexandru Cristian Cîndrea, Marius Militaru, Codrina Mihaela Levai, Sonia-Roxana Burtic, Ana Maria Pah, Laura Maria Craciun, Livia Ciolac, Tudor Rareș Olariu, Ovidiu Alexandru Mederle

**Affiliations:** 1 Research Center for Medical Communication, Victor Babes University of Medicine and Pharmacy, 300041 Timisoara, Romania; daian-ionel.popa@umft.ro (D.-I.P.); codrinalevai@umft.ro (C.M.L.); dr.soniaburtic@umft.ro (S.-R.B.); 2 Doctoral School, Faculty of General Medicine, Victor Babes University of Medicine and Pharmacy, 300041 Timisoara, Romania; williams.carmen@umft.ro (C.G.W.); alexandru.cindrea@umft.ro (A.C.C.); livia.ciolac@umft.ro (L.C.); 3 Emergency Municipal Clinical Hospital, 300254 Timisoara, Romania; marza.adina@umft.ro (A.M.M.); marius.militaru@umft.ro (M.M.); mederle.ovidiu@umft.ro (O.A.M.); 4 Department of Cardiology, Victor Babes University of Medicine and Pharmacy, E. Murgu Square No. 2, 300041 Timisoara, Romania; anamaria.pah@umft.ro (A.M.P.); laura.craciun@umft.ro (L.M.C.); 5 Department of Radiology, Victor Babes University of Medicine and Pharmacy, E. Murgu Square No. 2, 300041 Timisoara, Romania; aida.parvu@umft.ro; 6 Department of Functional Sciences, Victor Babes University of Medicine and Pharmacy, E. Murgu Square No. 2, 300041 Timisoara, Romania; atudor@umft.ro; 7 Department of Surgery, Emergency Discipline, Victor Babes University of Medicine and Pharmacy, 300041 Timisoara, Romania; dumitru.sutoi@umft.ro (D.S.); trebuian.cosmin@umft.ro (C.I.T.); 8 Department of Neuroscience, Discipline of Neurology II, Victor Babes University of Medicine and Pharmacy, E. Murgu Square No. 2, 300041 Timisoara, Romania; 9 Department of Infectious Diseases, Center for Diagnosis and Study of Parasitic Diseases, Victor Babes University of Medicine and Pharmacy, E. Murgu Square No. 1, 300041 Timisoara, Romania

**Keywords:** acute stroke, biomarkers, GFAP, UCH-L1, hemorrhagic acute stroke, ischemic acute stroke, emergency department, stroke mimics

## Abstract

**Background**: Rapid identification and treatment of stroke are essential for the patient. Our objective was to determine the diagnostic utility of glial fibrillary acidic protein (GFAP) and ubiquitin C-terminal hydrolase L1 (UCH-L1) in the emergency department to identify and differentiate acute stroke within 4.5 h of symptom onset in patients admitted with a stroke code alert. **Methods:** This study included 85 patients with a “code stroke alert” upon arrival at the emergency department. Individuals were grouped in two categories: patients with stroke (including 69 patients) and patients without stroke (including 16 patients). The research was conducted at the Emergency Municipal Clinical Hospital in Timișoara, Romania, the county’s second-largest hospital, which lacks a neurologist and a dedicated stroke unit. ** Results:** No significant differences were observed between the two groups (with stroke and without stroke) regarding most demographic or admission parameters. Significant differences were observed for the biomarkers GFAP (142.91 ± 102.19 pg/mL in patients with acute stroke vs. 37.76 ± 19.92 pg/mL in patients without stroke (*p* < 0.001)) and UCH-L1 (1307.68 ± 967.54 pg/mL in stroke patients vs. 189.81 ± 92.69 pg/mL in patients without stroke (*p* < 0.001)). Within the stroke group, 37 patients had acute ischemic stroke, while 32 patients were diagnosed with hemorrhagic stroke based on brain CT imaging. GFAP achieved an accuracy of 94.2% for differentiating hemorrhagic from ischemic stroke, with a cut-off value of 77.15 pg/mL. **Conclusions:** GFAP excellently differentiated acute stroke from stroke mimics, with high sensitivity, perfect specificity, and strong predictive values. Integrating GFAP and UCH-L1 measurements into emergency protocols may enhance stroke diagnosis, optimize patient triage, and ultimately improve outcomes by facilitating the faster initiation of appropriate therapies.

## 1. Introduction

Stroke represents a significant global health problem in terms of morbidity, disability, and mortality [1]. Therefore, rapid recognition and timely management of patients are vital. Acknowledging the significance of the initial 4.5 h in effective management of a stroke, the “Stroke Code” was developed. This code has been embraced by numerous countries globally. It is an organizational instrument designed to streamline the coordination between pre-hospital and hospital systems [2]. Its primary aim is to identify individuals who may be suitable, as this will considerably increase the number of suitable candidates for therapies such as thrombolysis and mechanical thrombectomy [3] while reducing transport duration and enhancing both pre-hospital and hospital diagnostic processes [4]. Both procedures are time-sensitive, as approximately 1.9 million neurons die in the brain per minute of an untreated stroke. Thrombolysis and thrombectomy should be performed within 4.5 h and up to 24 h of symptom onset. The earlier the treatment is given, the greater the chance of recovery [5].

In clinical practice, a significant proportion of patients who present with acute neurological symptoms are initially diagnosed with stroke but are experiencing what are known as stroke mimics—non-vascular conditions that imitate the clinical manifestations of a cerebrovascular event [6]. Stroke mimics encompass a wide range of disorders, such as seizures, migraines, hypoglycemia, functional (psychogenic) disorders, infections, or brain tumors, and are very common in the emergency department [7]. Approximately one-third of patients evaluated for suspected stroke are diagnosed with a condition other than stroke [8].

Accurate identification of these cases is crucial, as the inappropriate administration of thrombolytic therapy (e.g., alteplase) to a patient without an actual stroke can result in serious complications, including intracranial or systemic hemorrhage. Conversely, suppose thrombolytic treatment is delayed due to an incorrect suspicion of a stroke mimic in a patient who is experiencing an acute ischemic stroke. In that case, the optimal therapeutic window may be missed, significantly compromising neurological outcomes [6].

Therefore, the rapid and accurate differentiation between actual stroke and stroke mimics represents a major clinical challenge, with direct implications for therapeutic decisions and patient prognosis. This highlights the importance of rigorous diagnostic protocols, appropriate use of brain imaging, and neurologist expertise in evaluating patients with acute neurological deficits.

The identification of a stroke is primarily based on clinical presentation and neuroimaging, typically performed using computed tomography (CT) or magnetic resonance imaging (MRI). However, there is increasing interest in potential serum biomarkers as additional tools for early diagnosis [9]. As with other medical conditions, such as myocardial infarction, where blood biomarkers have been widely integrated into clinical management, a test for blood biomarkers linked to ischemic or hemorrhagic stroke could be an essential addition to currently available diagnostic techniques [10]. Two biomarkers recently examined in brain tissue injury are glial fibrillary acidic protein (GFAP) and ubiquitin C-terminal hydrolase L1 (UCH-L1). GFAP, a protein predominantly expressed in astrocytes, has very low serum concentrations in healthy individuals because it is not released under normal physiological conditions. In contrast, UCH-L1 is present in neurons and neuroendocrine cells, which play a crucial role in maintaining self-repair mechanisms [9].

However, it is not well studied whether GFAP and UCH-L1 can contribute to the clinical assessment of patients presenting with acute stroke symptoms in the emergency department. Therefore, it is of interest to examine this issue. The present study aimed to assess the diagnostic performance (sensitivity, specificity, predictive values, and accuracy) of these biomarkers in discriminating between stroke and non-stroke presentations, as well as between hemorrhagic and ischemic subtypes, within a clearly defined early time window (within 4.5 h of symptom onset). This study represents a challenge and could help distinguish patients with hemorrhagic stroke and identify and separate patients with acute neurological symptoms in cases of stroke mimic, acute hemorrhagic stroke, or acute ischemic stroke.

## 2. Materials and Methods

### 2.1. Study Design and Participants

This prospective observational diagnostic accuracy study involved patients who were identified with a “code stroke alert” in our emergency department at a hospital without a Neurological Department or a dedicated stroke unit. Conducted at the Emergency Municipal Clinical Hospital in Timisoara, Romania, the second-largest hospital in the county, the study was carried out between May 2024 and April 2025. During this period, 119 patients with a “code stroke alert” presented to our ED. This protocol was initiated when emergency medical services (EMS) personnel recognized a patient designated as an “acute stroke alert”, either during ambulance transport or by identification by the triage nurse or emergency physician upon arrival at the emergency department [11,12].

**Figure 1:**
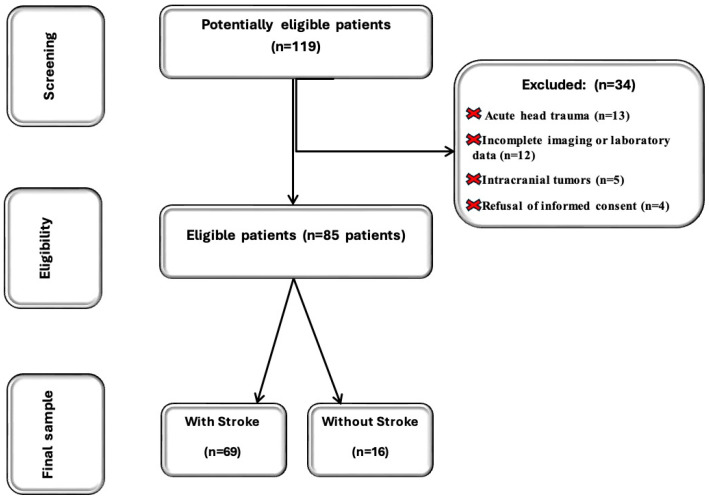
Subject screening flowchart.

As shown in the study flowchart in Figure 1, following a thorough screening process, 85 consecutive patients from the original cohort were deemed eligible for inclusion in our study; 69 were confirmed with acute stroke and included in the with-stroke group.

The group designated as without stroke comprised 16 patients who lacked a history of central nervous system disorders or, in the previous year, traumatic brain injuries. These individuals were not matched by age and sex to the acute stroke patients, as they were admitted under the acute stroke code and subsequently classified as stroke mimics following CT brain imaging and laboratory evaluations. A stroke mimic is characterized as a nonvascular condition that exhibits symptoms resembling those of a stroke, frequently making it difficult to differentiate from a genuine cerebrovascular accident. The detrimental side effects of the potential complications associated with thrombolytics, particularly bleeding, are significant; therefore, precise diagnosis is crucial [6]. However, their comorbidity burden was comparable to that of the with-stroke group. According to both clinical and imaging data, the inclusion criteria involved admission within six hours of symptom onset, the presence of neurological symptoms at the time of admission, and a confirmed stroke through radiological examination. The exclusion criteria were head trauma (n = 13), incomplete imaging or laboratory data (n = 12), intracranial tumors (n = 5), and refusal of informed consent (n = 4).

**Figure 2:**
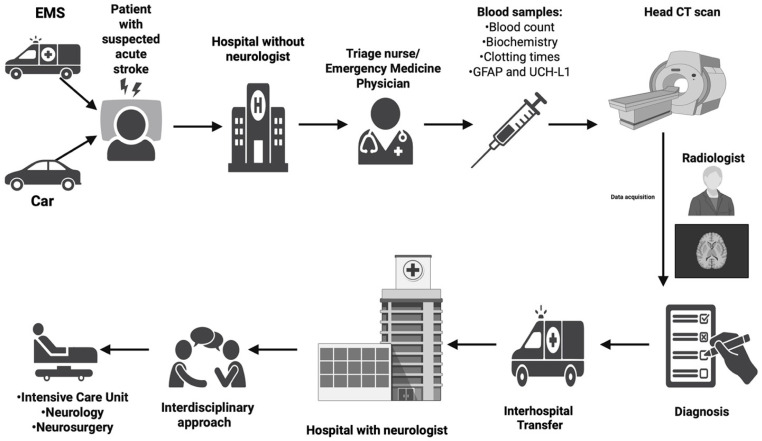
Our patient journey flow map (Created in BioRender. Popa, D. (2025) https://BioRender.com/3oy6t2g).

We mapped the pathway of our patients admitted with a “stroke code alert” in this study, starting with their admission to our ED. This included the collection of their laboratory tests and biomarkers, the performance of a brain CT scan, and the establishment of a diagnosis of acute stroke. Following this, the patient was transferred to a hospital with a neurologist and stroke unit, where an interdisciplinary approach (comprising a neurologist, radiologist, interventional radiologist, intensivist, and neurosurgeon) determined the therapeutic management of the patient and their admission to a specialized medical unit (Figure 2).

### 2.2. Blood Sample Collection and Processing

At the time of admission to the emergency department, blood samples were obtained from all study participants through venipuncture, ensuring that collection occurred no later than two and a half hours after the onset of symptoms, following standard hospital admission procedures. The collected blood samples were promptly transported to the hospital’s clinical laboratory, where they underwent centrifugation at 3000× *g* for 10 min. Following this process, the serum was divided into aliquots of 0.5 mL and stored at −20 °C, adhering to the manufacturer’s guidelines that stipulate a maximum storage duration of one month at this temperature. Upon identifying patients diagnosed with acute stroke, the serum samples were retrieved from storage and assigned to two distinct groups. Samples from individuals who did not meet the inclusion criteria were excluded from the analysis. The processing of serum samples was conducted by board-certified laboratory technicians in the hospital’s clinical laboratory, who were blinded to the clinical data. The quantification of UCH-L1 and GFAP serum levels was performed using the Abbott kit (04W1701, Abbott, Abbott Park, IL, USA), produced by Abbott in Sligo, Ireland, with each measurement executed in full calibration mode. The Alinity i test is a series of in vitro diagnostic chemiluminescent microparticle immunoassays (CMIA) designed for the quantitative assessment of GFAP and UCH-L1 levels in both plasma and serum. It offers a semi-quantitative interpretation of results based on a combination of these measurements. The Alinity i platform, developed by Abbott Laboratories in Sligo, Ireland, serves as an automated immunoassay analyzer for clinical laboratories, boasting a throughput capability of up to 200 tests per hour and accommodating 47 assay reagent cartridges per module. This test comprises two separate immunoassays for GFAP and UCH-L1, which are analyzed using a single plasma sample. Our samples were measured in duplicates. The results for GFAP and UCH-L1 are reported individually.

The sample analysis duration was approximately 18 min, during which the concentrations of the two biomarkers were displayed on the screen. Each assay’s reportable range spanned from the limit of detection (LoD) to the upper limit of quantitation (LoQ). The reportable range for GFAP extended from 3.2 pg/mL to 42,000 pg/mL, while for UCH-L1, it spanned from 18.3 pg/mL to 25,000 pg/mL. Measurements that exceeded this reportable range did not yield a value on the Alinity i platform. The lower limit of quantification (LoQ) was established at 6.1 pg/mL for GFAP and at 26.3 pg/mL for UCH-L1. The analytical measuring interval (AMI) was defined by the spectrum of values that exhibited acceptable performance in terms of linearity, imprecision, and bias. For GFAP, the AMI ranged from 6.1 pg/mL to 42,000 pg/mL, whereas for UCH-L1, it extended from 26.3 pg/mL to 25,000 pg/mL According to the instructions for use, the reproducibility ranged from a 1.7% coefficient of variation (CV) to a 4.7% CV for GFAP and from a 2.7% CV to a 5.6% CV for UCH-L1.

The criteria established by the manufacturer guided the testing, quality controls, and interpretation of the laboratory test results. The cut-off values were determined by optimizing both sensitivity and specificity. For the purpose of this study, the laboratory results for these biomarkers were not available to the doctors on duty, laboratory technicians, or radiologists.

### 2.3. Radiological Assessment

Upon admission to the emergency department, all patients who were enrolled underwent immediate neuroimaging following the “code stroke alert” protocol.

The imaging protocol was standardized in accordance with the recommendations of our national acute stroke protocol [11]. Non-contrast brain computed tomography (CT) utilizing a 128-slice CT Siemens Somatom X cite syngo CT VA 40 from Erlangen, Germany was the primary imaging modality used in all patients to exclude intracranial hemorrhage, identify early signs of ischemic infarction (e.g., loss of gray-white matter differentiation, sulcal effacement), and detect mass lesions or other structural abnormalities. Only in selected cases where the initial findings were inconclusive or clinical suspicion of large vessel occlusion (LVO) was high, CT angiography (CTA) of the head and neck vessels was performed to assess for arterial occlusions or stenosis, dissections, and vascular anomalies. Magnetic resonance imaging (MRI) was not used in the acute diagnostic phase at our ED due to unavailability and time constraints in the emergency setting.

All imaging studies were interpreted by on-call radiologists, and in stroke-confirmed cases, further evaluation was conducted at the referral hospital with a dedicated neurology and stroke team.

### 2.4. Statistical Analysis

Data analysis was performed with JASP v0.19.3 (open-source statistical analysis software supported by the University of Amsterdam). The mean and standard deviation, median, and interquartile range were used to present numerical variables, while frequency and percentages were used for nominal variables. The distribution of continuous variables was assessed using the Shapiro–Wilk test to determine the normality of the numerical data distribution. Mann–Whitney U and chi-squared tests were used to compare the characteristics of patients with and without stroke. Also, the two proteins, GFAP and UCH-L1, were analyzed as classifiers for stroke and hemorrhagic stroke. The results are presented with 95% confidence intervals. A *p*-value of less than 0.05 is considered statistically significant. The cut-off values were established by maximizing sensitivity + specificity.

To control for type I error inflation resulting from multiple comparisons, we applied the Benjamini–Hochberg method (1995) to adjust the *p*-values.

## 3. Results

### 3.1. Demographic Characteristics

The study comprised a total of 85 participants, including 69 individuals diagnosed with acute stroke (designated as the stroke group) and 16 individuals presenting with stroke mimics (referred to as the without-stroke group). Within the cohort of acute stroke patients, brain CT imaging revealed that 37 individuals had acute ischemic stroke, while 32 were diagnosed with hemorrhagic stroke. The baseline characteristics of these participants—encompassing age, sex, residence, mode and time of arrival, as well as systolic and diastolic blood pressure (SBP and DBP), Glasgow Coma Scale (GCS) scores, and neurological deficits classified according to the National Institutes of Health Stroke Scale (NIHSS)—are detailed in Table 1.

The laboratory test results for the acute stroke patients are provided in Table 2. No statistically significant differences were observed between the groups regarding hemoglobin (*p* = 0.053), platelet count (*p* = 0.727), blood glucose (*p* = 0.430), INR (*p* = 0.213), prothrombin time (*p* = 0.187), or partial thromboplastin time (*p* = 0.135). There were statistically significant differences between the groups in the analyses of GFAP (142.91 ± 102.19 pg/mL versus 37.76 ± 19.92 pg/mL, *p* < 0.001) and UCH-L1 (1307.68 ± 967.54 pg/mL versus 189.81 ± 92.69 pg/mL, *p* < 0.001).

Regarding the symptoms patients presented at admission in the ED, no statistically significant differences were observed between the groups, nor were there any differences in comorbidities (Table 3).

There were no significant differences between the two groups, ischemic stroke versus hemorrhagic stroke, regarding comorbidities based on Fisher’s chi-squared test (Table 4).

Table 5 show that the outcomes between the two groups were an increased number of hospitalization days for patients with stroke vs. those without stroke (13.99 ± 6.06 days vs. 5.31 ± 4.28 days, *p* < 0.001).

### 3.2. Diagnostic Performance of GFAP and UCH-L1

**Figure 3:**
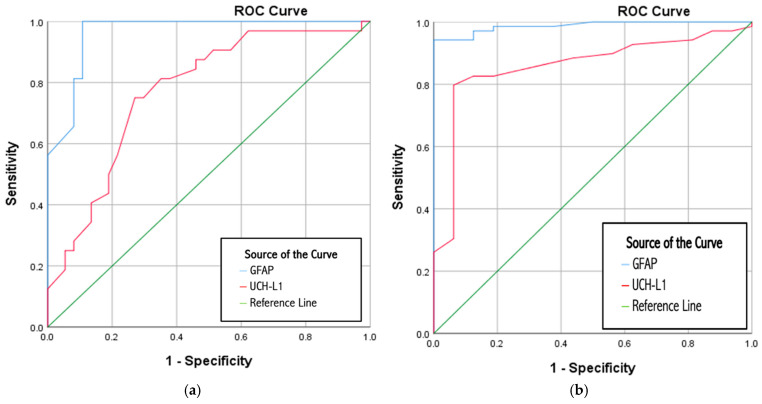
ROC curves for predictive performance of GFAP and UCH-L1 for diagnostic acute stroke vs. without stroke (**a**) and for hemorrhagic acute stroke vs. ischemic acute stroke (**b**).

A ROC curve analysis was performed to analyze the predictive performance of biomarkers GFAP and UCH-L1 for the diagnosis of acute stroke (Table 6 and Figure 3). We determined the predictive value of these two biomarkers by analyzing the patients from the group with stroke (n = 69) vs. the patients without stroke (n = 16), and the patients with acute ischemic stroke (n = 37) vs. the patients with hemorrhagic stroke (n = 32) from the group with stroke. When the with-stroke vs. the without-stroke groups were analyzed, the performance of GFAP in predicting acute stroke was excellent, and the UCH-L1 was very good. GFAP is a significant classifier (*p* < 0.001) of stroke, with a sensitivity of 94.2%, specificity of 100%, positive predictive value of 100%, negative predictive value of 80%, and accuracy of 95.3%. UCH-L1 is a significant classifier (*p* < 0.001) of acute stroke, with a sensitivity of 79.7%, specificity of 93.8%, positive predictive value of 98.2%, negative predictive value of 51.7%, and accuracy of 82.4%. The diagnostic accuracy of GFAP as a biomarker showed a 94.2% accuracy in discriminating between hemorrhagic vs. ischemic acute stroke with a cut-off value of 77.15 pg/mL. Furthermore, UCH-L1 as a biomarker was accurate in approximately 73.9% of hemorrhagic versus ischemic acute discrimination cases, with a cut-off value of 897.85 pg/mL.

The NPV was significantly lower, reflecting the possibility of a high proportion of positive cases (patients with stroke) in the sample. UCH-L1 has very high sensitivity and specificity, but its NPV is affected by the distribution of cases (many strokes vs. few without stroke). UCH-L1 provides excellent sensitivity and negative predictive value for identifying hemorrhagic stroke, but its PPV remains slightly below 90%, with a relatively wide confidence interval.

Power calculations were carried out to assess whether the sample size was adequate.

A post hoc power analysis was performed using G*Power. Comparing the proportion of GFAP-positive cases between stroke patients (79.7%) and patients without stroke (6.25%), the resulting power was 99%, even for an adjusted significance level of α = 0.02. This value suggests that the total sample (n = 85), despite the imbalance between groups (69 vs. 16), has sufficient power to detect significant differences between groups, thereby reducing the risk of a Type II error. However, the results for the without-stroke group, composed of only 16 patients, should be interpreted with caution in terms of the stability of the specificity and NPV estimates, as reflected in the width of the corresponding confidence intervals.

For the comparison of patients with stroke versus those without stroke using the UCH-L1 biomarker, a post hoc power analysis (performed with G*Power) showed 100% power, even at a stringent significance level (α ≈ 0). The marked difference between the UCH-L1 positivity rates (94.2% in the group with stroke vs. 0% in the group without stroke) was so pronounced that the statistical test had a negligible risk of type I or type II error. This finding supports the robustness of the conclusions regarding the diagnostic performance of UCH-L1-based diagnostics in differentiating stroke from non-stroke cases.

In the comparison of hemorrhagic and ischemic stroke based on GFAP status (positive vs. negative), post hoc power analysis using G*Power showed a power of 94.9% for an observed difference of 75.0% vs. 27.0% GFAP +, at a significance level of α = 0.033. These values indicate a low risk of type II error and support the validity of the difference between the two groups in terms of GFAP biomarker expression. However, due to the relatively small number of patients in each subgroup (n = 32 for hemorrhagic and n = 37 for ischemic), the estimates should be interpreted in the context of moderate confidence intervals, which justifies the need for replication studies on larger samples.

For the comparison of hemorrhagic vs. ischemic stroke based on UCH-L1 biomarker expression, post hoc analysis in G*Power showed a statistical power of 92.9% and a significance level of α = 0.032. These results suggest that the detected difference (100% vs. 10.8% UCH-L1 positive) is statistically robust, with a low risk of type I and type II errors. They also support the promising nature of the biomarker in discriminating between stroke types.

To control for the inflation of type I errors resulting from multiple comparisons, we applied the Benjamini–Hochberg (1995) method for adjusting *p*-values. After correction, GFAP and UCH-L1 remained significantly different between the stroke and without-stroke groups (adjusted *p* < 0.01), supporting their relevance as potential diagnostic biomarkers. The other variables are not significant, either before or after correction (Table 7).

## 4. Discussion

The primary findings of this study indicate that patients experiencing an acute stroke who were identified with a “stroke alert” exhibited significantly elevated serum levels of both GFAP and UCH-L1 when compared to those presenting with stroke mimics (the group without stroke). Furthermore, serum GFAP demonstrated a diagnostic accuracy of 94.2% in differentiating between acute hemorrhagic stroke and acute ischemic stroke, utilizing a cut-off value of 77.15 pg/mL. Additionally, our findings further substantiate that circulating GFAP possesses considerable discriminatory capability for the clinically significant differential diagnosis of acute hemorrhagic stroke versus acute ischemic stroke. In contrast, UCH-L1 showed an accuracy of approximately 73.9% in distinguishing between hemorrhagic and ischemic strokes, with a cut-off value of 897.85 pg/mL in this context.

Our results are consistent with the data from the literature.

Fewer than 10 other studies have examined both biomarkers in acute stroke [9,13,14,15,16]. A recent investigation by Kraljević et al., from Croatia, examined the potential application of specific biomarkers in differentiating large vessel occlusion (LVO) from small vessel occlusion (SVO) in patients presenting to the neurology department with a sudden onset of focal neurological deficits. The study identified significant variations in serum levels of GFAP and UCH-L1 across all groups, including the control, SVO, and LVO (GFAP: 30.19 pg/mL for the control, 58.6 pg/mL for SVO, and 321.3 pg/mL for LVO; UCH-L1: 117.7 pg/mL for the control, 251.8 pg/mL for SVO, and 573.1 pg/mL for LVO; *p* < 0.0001), with LVO exhibiting the highest concentrations. The authors concluded that utilizing a combination of GFAP and UCH-L1 may serve as an effective diagnostic tool for distinguishing between LVO and SVO in patients experiencing acute ischemic stroke [13]. Another investigation involving 251 patients (mean age [± SD] 72 ± 15 years; including 5 cases of intracerebral hemorrhage (ICH), 23 cases of ischemic stroke, and 14 cases of stroke mimics in the prehospital phase; along with 59 cases of ICH, 148 ischemic strokes, and 2 cases of stroke mimics in the hospital phase) revealed that GFAP and UCH-L1, when assessed by a rapid biological test, were significantly elevated in patients with ICH compared to those with other conditions. This finding highlights the potential of these two biomarkers in differentiating ICH from ischemic strokes and stroke mimics [14].

Over a two-year period, an observational group from China, consisting of 80 patients diagnosed with acute cerebral infarction, was formed and compared with a control group of 80 healthy individuals. The expression levels of UCH-L1 and GFAP in the observation group were found to be significantly higher than those in the control group. However, no statistically significant differences were observed between the two groups in the UCH-L1 and GFAP levels at different time points. The sensitivity and specificity for UCH-L1 and GFAP were recorded at 75.0% and 87.5% and at 81.3% and 90.0%, respectively. The areas under the receiver operating characteristic curves for UCH-L1 and GFAP were 0.670 and 0.757, respectively. No significant differences were observed in age, gender, alcohol consumption, smoking habits, diabetes, or hyperlipidemia between the two groups. However, the rate of high blood pressure in the observation group was significantly higher than in the control group. The Spearman/Pearson analysis revealed a positive correlation between the serum levels of UCH-L1 and GFAP in individuals with hypertension. In contrast, a negative correlation was noted with sex, age, diabetes, hyperlipidemia, alcohol intake, smoking, and other variables. The general data collected at various times within the observation group did not show statistically significant differences [16]. The baseline characteristics of our study participants—encompassing age, sex, residence, mode and time of arrival, as well as diastolic blood pressure, Glasgow Coma Scale scores, and NIHSS (detailed in Table 1)—present no significant differences between the with-stroke and without-stroke groups. Only the systolic blood pressure in the with-stroke group was significantly higher than in the without-stroke group (*p* < 0.001).

In a similar study, the two biomarkers were assessed within 4.5 h of symptom onset but using units of measurement in nanograms per milliliter (ng/mL). The concentrations of both UCH-L1 and GFAP were found to be significantly increased in patients with intracerebral hemorrhage compared to those in the control group (*p* < 0.0001). However, it is noteworthy that only GFAP showed a significant difference when comparing ICH with ischemic acute stroke (*p* < 0.0001). The area under the curve (AUC) for GFAP in distinguishing between ICH and IS was reported to be 0.86, with a sensitivity of 61% and a specificity of 96% at a threshold of 0.34 ng/mL [15]. GFAP and UCH-L1 demonstrated a significant correlation with the dispersion of blood volume, indicating their direct influence on brain damage, as assessed in patients experiencing spontaneous subarachnoid hemorrhage at 24 h, 72 h, and one week following the hemorrhagic event [17]. These findings, consistent with ours, indicate that both UCH-L1 and GFAP levels in the plasma increase shortly after stroke and that distinct biomarker release profiles are related to stroke characteristics and type.

In a separate study spanning three years and involving patients exhibiting symptoms of suspected stroke lasting ≤ 4.5 h, GFAP was assessed in a total of 299 individuals (44% diagnosed with acute ischemic stroke, 38% presenting stroke mimics, 10% with intracranial hemorrhage, and 7% with transient ischemic attack). The sensitivity of GFAP in identifying patients with hemorrhagic stroke was found to be 25.0% (95% CI 11.5–43.4), while its specificity reached 100.0% (95% CI 98.6–100.0). Additionally, detecting patients with large vessel occlusion yielded a sensitivity of 55.6% (95% CI 35.3–74.5) and a specificity of 82.4% (95% CI 77.3–86.7) [18]. In our study, the accuracy of GFAP as a biomarker showed 94.2% accuracy in distinguishing between hemorrhagic and ischemic stroke, with a cut-off value of 77.15 pg/mL, with 100% sensitivity, 89.2% specificity, and 100% negative predictive value, demonstrating that serum GFAP has diagnostic value in distinguishing between hemorrhagic and ischemic stroke. Specificity of 89.2%, positive predictive value of 88.9%, and negative predictive value of 100%, demonstrating that serum GFAP has promising diagnostic value for detecting intracranial hemorrhage in patients with acute stroke.

In a study involving 35 patients diagnosed with acute ischemic stroke, which included 10 individuals with large vessel occlusion, serum GFAP levels were evaluated alongside cases of intracerebral hemorrhage (n = 12), transient ischemic attack (n = 4), and stroke-like symptoms (n = 11). The findings indicated that GFAP levels may serve as a valuable adjunctive tool for differentiating between intracerebral hemorrhage and acute ischemic stroke. Furthermore, concerning acute ischemic stroke, GFAP may yield insights regarding the duration from onset and the extent of ischemic tissue damage observable through neuroimaging, particularly in instances of stroke with large vessel occlusion [19].

It is recognized that the “last-seen-well time” following the onset of symptoms is crucial for accurately interpreting the levels of these biomarkers. Existing studies have indicated that very early sampling (within one hour) is not only feasible but also captures significant elevations in both biomarkers. An ultra-early investigation conducted with a mobile stroke unit [20] demonstrated that the median time for blood sampling was 58 min post-symptom onset (ranging from 36 to 133 min), revealing that UCH-L1 and GFAP concentrations increased by as much as tenfold above normal values within just 36 min. This finding underscores the rapid rise of both biomarkers during acute stroke events. Additionally, Paul et al. observed that in patients with acute ischemic stroke and a known stroke onset time of less than 4.5 h (n = 12), GFAP levels appeared to elevate with delayed symptom onset [19]. These preliminary findings are deemed worthy of further investigation.

As we have already shown, this study provides compelling evidence supporting the diagnostic utility of serum GFAP and UCH-L1 in the acute evaluation of patients with suspected stroke. Patients admitted with a “stroke alert” exhibited significantly higher serum concentrations of both GFAP and UCH-L1 compared to those with stroke mimics, underscoring their potential role in the early differentiation of actual stroke cases.

In the case of stroke chameleons, where stroke presents atypically and can be confused with other conditions, the use of biomarkers, such as GFAP and UCH-L1, can help differentiate between a real stroke and a stroke mimic even in the emergency department [21]. For example, elevated GFAP levels may indicate a hemorrhagic stroke, while low levels may suggest the absence of such a stroke. This information can guide treatment decisions and prevent the unnecessary administration of thrombolytics or, conversely, delay treatment in cases of actual stroke [22].

Furthermore, in our study, GFAP demonstrated superior diagnostic accuracy in distinguishing between acute hemorrhagic stroke and acute ischemic stroke, with an accuracy of 94.2% at a cut-off value of 77.15 pg/mL, accompanied by excellent sensitivity (100%) and negative predictive value (100%). This finding affirms GFAP as a reliable biomarker for ruling out intracranial hemorrhage in emergencies. In contrast, UCH-L1 displayed moderate diagnostic performance (accuracy of 73.9% at a cut-off of 897.85 pg/mL), suggesting a complementary, though less definitive, role in stroke subtyping. Our results are consistent with the existing literature, highlighting elevated GFAP and UCH-L1 levels in hemorrhagic stroke versus ischemic stroke, and differentiating between large and small vessel occlusions. Importantly, our study is among the first to concurrently analyze these biomarkers in stroke and non-stroke presentations within a real-world emergency department context.

Overall, the findings support the clinical applicability of serum GFAP and, to a lesser extent, UCH-L1 as valuable diagnostic tools for the early and accurate classification of stroke type. This will thereby inform timely therapeutic decisions and optimize acute stroke management.

## 5. Study Limitations and Further Directions

As a limitation, this study is a monocentric observational study with a small sample size. Therefore, the preliminary results should be regarded solely as hypothesis-generating, necessitating validation in larger cohorts admitted to emergency departments. Conducted in a single emergency department without the involvement of a neurologist or a dedicated stroke unit, the findings may not be representative of the broader population due to the unique characteristics of the studied group. Biomarker levels were measured only upon admission to the emergency department; serial assessments could have yielded further insights into stroke progression and biomarker kinetics. Another limitation of this study lies in the inability to utilize TOAST classification for the subtyping of ischemic strokes. Due to the transfer of patients to a stroke center after their initial evaluation in the emergency department, comprehensive follow-up data and advanced neurovascular imaging (such as CTA and MRI) were not consistently accessible.

Our findings align with earlier studies that indicate GFAP is a reliable biomarker for differentiating between acute stroke and other conditions, demonstrating comparable diagnostic accuracy. Nevertheless, considerable variability exists in the diagnostic thresholds reported across studies, as well as discrepancies in units of measurement (ng/mL or pg/mL), which may raise concerns regarding clinical applicability. Several factors may contribute to this inconsistency, including patient demographics, variations in sample types (plasma versus serum), differences in sampling times, and a lack of standardization. Additionally, analytical factors such as batch-to-batch variability in ELISA kits, equipment, laboratory practices, and the absence of validated reference methods contribute to this issue. To establish and unify optimal reference values, thorough and standardized quality control studies are essential. Further research will be required to address these issues and understand the generalizability of our study’s results.

## 6. Conclusions

Our research indicates that GFAP is an exceptionally reliable biomarker for differentiating acute stroke from stroke mimics, demonstrating outstanding sensitivity, perfect specificity, and robust positive and negative predictive values.

These findings suggest that the combined evaluation of GFAP and UCH-L1 may have potential as diagnostic tools in the early differentiation of stroke subtypes, particularly in emergency settings with limited access to advanced imaging. Large multicenter studies are needed to confirm these findings.

Incorporating the analysis of these biomarkers into standard emergency protocols could improve the initial diagnostic procedure and optimize patient triage, prioritize essential resources, such as imaging and interventions, and enable the quicker initiation of suitable, targeted therapies. Ultimately, this integration could improve short- and long-term clinical outcomes by reducing treatment delays and the risk of misdiagnosis in patients with acute neurological symptoms.

## Figures and Tables

**Table 1:** Baseline characteristics of study patients (n = 85).

Association Variables For Numerical: Mean ± SD; Median (Q1–Q3) For Nominal: n (%)		Study Participants		*p* Value
		With Stroke (n = 69)	Without Stroke (n = 16)	
Patient characteristics				
Age, years		70.29 ± 12.17 73 (63–81)	73.31 ± 8.67 74 (63.5–80.3)	0.558
SBP, mmHg		144.93 ± 25.43 150 (135–155)	164.38 ± 13.65 160 (155–175)	0.001 *
DBP, mmHg		81.01 ± 17.38 80 (70–95)	85 ± 10.8 82.5 (80–91.3)	0.463
GCS		13.32 ± 1.71 13 (12–15)	13.88 ± 1.67 15 (12–15)	0.245
NIHSS at presentation		18.8 ± 7.13 18 (14–23)	7.25 ± 2.21 7 (5–9)	<0.001 *
Gender	Male	27 (79.4%)	7 (20.6%)	0.734
	Female	42 (82.4%)	9 (17.6%)	
Residence	Urban	43 (84.3%)	8 (15.7%)	0.365
	Rural	26 (76.5%)	8 (23.5%)	
Shift	Day	63 (82.9%)	13 (17.1%)	0.239
	Night	6 (66.7%)	3 (33.3%)	
Arrival mode	EMS	51 (82.3%)	11 (17.7%)	0.675
	Walk-in	18 (78.3%)	5 (21.7%)	

* Significant difference. SBP, systolic blood pressure; DBP, diastolic blood pressure; GCS, Glasgow Coma Score; NIHSS, National Institutes of Health Stroke Scale; n, number of patients; SD, standard deviation; Q1, quartile 1; Q3, quartile 3.

**Table 2:** Blood sample test results of investigated patients (n = 85).

Association Variables For Numerical: Mean ± SD; Median (Q1–Q3) For Nominal: n (%)	Study Participants		*p* Value
	With Stroke (n = 69)	Without Stroke (n = 16)	
Blood test sample results			
Hemoglobin, g/dL	13.2 ± 2.26 13.8 (10.7–14.7)	14.92 ± 2.57 14.1 (13.7–15.7)	0.053
Platelets count, ×109 μL	261.52 ± 90.77 214 (196–339)	317.81 ± 187.75 213.5 (201.3–362.3)	0.727
Blood glucose, mg/dL	145.57 ± 67.08 126 (97–178)	148.19 ± 51.83 124.5 (114.3–183.5)	0.430
INR	1.01 ± 0.13 1 (0.9–1.1)	1.04 ± 0.12 1 (1–1.1)	0.213
Prothrombin time, seconds	12.7 ± 1.41 12.6 (11.7–13.1)	13.03 ± 1.29 13 (12.1–13.3)	0.187
Partial thromboplastin time, seconds	24.05 ± 2.51 23.3 (22.7–24.2)	24.81 ± 2.35 24.8 (23.2–27.1)	0.135
GFAP	142.91 ± 102.19 76.1 (43.5–136.2)	37.76 ± 19.92 38.2 (32.1–38.8)	<0.001 *
UCH-L1	1307.68 ± 967.54 1123.2 (561.3–1604.9)	189.81 ± 92.69 157.8 (122.8–202.8)	<0.001 *

* Significant difference. INR, international normalized ratio; GFAP, glial fibrillary acidic protein; UCH-L1, ubiquitin carboxy-terminal hydrolase L1. Reference values: hemoglobin (12–15 g/dL); platelet count (150–410/×10^9^ μL)*;* glucose (74–106 mg/dL); INR (0.8–1.2/INR); prothrombin time (9.9–12.3/s); partial prothromboplastin time (21.6–28.7/s).

**Table 3:** Acute stroke symptoms at admission in the ED and comorbidities for all patients (n = 85).

Association Variables For Numerical: Mean ± SD; Median (Q1–Q3) For Nominal: n (%)		Study Participants		*p* Value
		With Stroke (n = 69)	Without Stroke (n = 16)	
Acute stroke symptoms				
Hemiparesis	Left	47 (82.5%)	10 (17.5%)	0.667
	Right	22 (78.6%)	6 (21.4%)	
Aphasia		50 (79.4%)	13 (20.6%)	0.470
Dysarthria		29 (80.6%)	7 (19.4%)	0.900
Headache		27 (77.1%)	8 (22.9%)	0.426
Fatigue		49 (79.0%)	13 (21.0%)	0.406
Facial paresis/palsy		35 (81.4%)	8 (18.6%)	0.958
Loss of balance		48 (78.7%)	13 (21.3%)	0.350
Blindness		14 (82.4%)	3 (17.6%)	0.890
Comorbidities				
Hypertension		45 (83.3%)	9 (16.7%)	0.569
Diabetes mellitus		49 (80.3%)	12 (19.7%)	0.750
Obesity		39 (78.0%)	11 (22.0%)	0.371
Previously stroke		35 (79.5%)	9 (20.5%)	0.690
Atrial Fibrillation		43 (81.1%)	10 (18.9%)	0.989
Anticoagulation treatment		10 (66.7%)	5 (33.3%)	0.989

**Table 4:** Comorbidities for all patients (n = 85).

Association Variables n (%)	Study Participants		*p* Value
	Ischemic Stroke (n = 37)	Hemorrhagic Stroke (n = 32)	
Comorbidities			
Hypertension	37 (53.6%)	32 (46.4%)	0.499
Diabetes mellitus	29 (59.2%)	20 (40.8%)	0.187
Obesity	24 (61.5%)	15 (38.5%)	0.152
Previously stroke	22 (62.9%)	13 (37.1%)	0.150
Atrial Fibrillation	25 (58.1%)	18 (41.9%)	0.455

**Table 5:** Outcomes of the patients (n = 85).

Association Variables For numerical: Mean ± SD; Median (Q1–Q3) For Nominal: n (%)		Study Participants		*p* Value
		With Stroke (n = 69)	Without Stroke (n = 16)	
Outcome				
Hospitalization, days		13.99 ± 6.06 13 (10–18)	5.31 ± 4.28 3 (2.5–6.5)	<0.001 *
Outcome	live	55 (77.5%)	16 (22.5%)	0.050
	death	14 (100.0%)	0 (0.0%)	

* Significant difference.

**Table 6:** Predictive performance of GFAP and UCH-L1 for the diagnosis of acute stroke.

Biomarker	GFAP		UCH-L1	
Sample	With Stroke vs. Without Stroke	Hemorrhagic vs. Ischemic	With Stroke vs. Without Stroke	Hemorrhagic vs. Ischemic
Cut-off (pg/mL)	41.3	77.15	397.85	897.85
AUC (95% CI)	0.953 (0.97, 1)	0.942 (0.924, 1)	0.824 (0.763, 0.956)	0.739 (0.659, 0.883)
SN% (95% CI)	79.7 (68.3–88.4)	75.0 (56.6–88.5)	94.2 (85.8–98.4)	100 (89.1–100)
SP% (95% CI)	93.8 (69.8–99.8)	73.0 (55.9–86.2)	100 (79.4–100)	89.2 (74.6–97.0)
PPV% (95% CI)	98.2 (90.4–99.9)	70.6 (52.5–84.9)	100 (94.5–100)	88.9 (73.9–96.9)
NPV% (95% CI)	51.7 (32.5–70.6)	77.1 (59.9–89.6)	80.0 (56.3–94.3)	100 (89.4–100)

AUC—area under the curve; SN—sensitivity; SP—specificity; PPV—positive predictive value; NPV—negative predictive value.

**Table 7:** Estimated results for false discovery rate (FDR) (Benjamini–Hochberg).

Variable	*p* Raw	*p* Adjusted (BH)
Hemoglobin, g/dL	0.053	0.085
Platelets count, ×109 μL	0.727	0.727
Blood glucose, mg/dL	0.430	0.574
INR	0.213	0.341
Prothrombin time, s	0.187	0.311
Partial thromboplastin time, s	0.135	0.248
GFAP	<0.001	0.008
UCH-L1	<0.001	0.008

## Data Availability

The datasets are not publicly available, but de-identified data may be provided upon request from Florina Buleu.
